# Supplementary material for: NFAT activation by FKBP52 promotes cancer cell proliferation by suppressing p53
Source: Life Sci Alliance. 2024 May 21;7(8):e202302426. doi: 10.26508/lsa.202302426 (PMC11109481; doi:10.26508/lsa.202302426)
Supplement: Supplementary file 2 [file LSA-2023-02426_TableS2.docx]

**Table S2 Sequences of shRNA**

| **Gene** | **Sequence (5’-3’)** |
| --- | --- |
| Luciferase | CGTGCGTGGAATGCTTCGA |
| FKBP52-1 | GCGGAATCATTCGCAGAATAC |
| FKBP52-2 | GCAAGGACAAATTCTCCTTTG |
| FKBP52-3 | GCATGGAGAAAGGAGAACATT |
| FKBP52-4 | GAAGAAGCTCTATGCCAATAT |
| p53 | GACTCCAGTGGTAATCTACT |
| Calcineurin Aα-1 | GCCAAGGGCTTAGACCGAATT |
| Calcineurin Aα-2 | GGGTCAGAAGAAGATGGATTT |
| NFATc1-1 | GCCAGTACCAGCGTTTCA |
| NFATc1-2 | CGACATTGAACTTCGGAAA |
| NFATc2-1 | CATTAAACAGGAGCAGAA |
| NFATc2-2 | TCCTTAAGCCGCACGCCTT |
| NFATc3-1 | GCTTACCACATCATGGATTAC |
| NFATc3-2 | GGAAAGGCCTTCTAGAGATCA |
