# Supplementary material for: NFAT activation by FKBP52 promotes cancer cell proliferation by suppressing p53
Source: Life Sci Alliance. 2024 May 21;7(8):e202302426. doi: 10.26508/lsa.202302426 (PMC11109481; doi:10.26508/lsa.202302426)
Supplement: Supplementary file 3 [file LSA-2023-02426_TableS3.docx]

**Table S3 Antibodies**

| **Antigen** | **Application** | **Catalog No.** | **Company** |
| --- | --- | --- | --- |
| β-actin | WB | ab6276 | Abcam |
| HSP90 | WB | sc-13119 | Santa Cruz |
| FKBP52 | WB, IF | 10655-1-AP | Proteintech |
| FKBP52 | ChIP | 66040-2-Ig | Proteintech |
| p53 | WB | sc-126 | Santa Cruz |
| p21 | WB | sc-6246 | Santa Cruz |
| PUMA | WB | sc-374223 | Santa Cruz |
| GADD45A | WB | cs4632 | Cell signaling |
| Calcineurin Aα | WB | ab52761 | Abcam |
| NFATc1 | WB, ChIP | sc-7294 | Santa Cruz |
| NFATc2 | WB | sc-7296 | Santa Cruz |
| NFATc3 | WB, ChIP | 18222-1-AP | Proteintech |
| MDM2 | WB | GTX100531 | Gene Tex |
| HDAC2 | WB | sc-7899 | Santa Cruz |
| H3 | WB | MABI0301 | Wako |
| γH2AX | WB | 05-636 | Merck Millipore |
| H2AX | WB | sc-517336 | Santa Cruz |
| FLAG | WB | M185-3L | MBL |
| HA | WB, IP | ab9110 | Abcam |
| V5 | IF | R960-25 | Thermo Fisher |
