# Supplementary material for: NFAT activation by FKBP52 promotes cancer cell proliferation by suppressing p53
Source: Life Sci Alliance. 2024 May 21;7(8):e202302426. doi: 10.26508/lsa.202302426 (PMC11109481; doi:10.26508/lsa.202302426)
Supplement: Supplementary file 4 [file LSA-2023-02426_TableS4.docx]

**Table S4 Sequences of primer for qPCR**

| **Target Gene** | **Forward primer (5’-3’)** | **Reverse primer (5’-3’)** |
| --- | --- | --- |
| *ACTB* | AGAAAATCTGGCACCACACC | AGAGGCGTACAGGGATAGCA |
| *GAPDH* | GAGTCAACGGATTTGGTCGT | TTGATTTTGGAGGGATCTCG |
| *TBP* | TGTATCCACAGTGAATCTTGGTTG | GGTTCGTGGCTCTCTTATCCTC |
| *TP53* | CCTCAGCATCTTATCCGAGTGG | TGGATGGTGGTACAGTCAGAGC |
| *p21*(*CDKN1A*) | TTAGCAGCGGAACAAGGAGT | GCCGAGAGAAAACAGTCCAG |
| *GADD45A* | CTGGAGGAAGTGCTCAGCAAAG | AGAGCCACATCTCTGTCGTCGT |
| *PUMA* (*BBC3*) | ACGACCTCAACGCACAGTACGA | CCTAATTGGGCTCCATCTCGGG |
| *MDM2* | TGTTTGGCGTGCCAAGCTTCTC | CACAGATGTACCTGAGTCCGATG |
| MDM2 (ChIP) | ACGGTAACCAGCATCTTCGT | CCGCTGGAGTTGTACCCAAA |
